# Blood Rheology and Platelet Function in Untreated Early-Stage Essential Hypertensives Complicated with Metabolic Syndrome

**DOI:** 10.1155/2012/109830

**Published:** 2012-04-18

**Authors:** Hiroko Sugimori, Fumihiro Tomoda, Tsutomu Koike, Hiroyuki Kinuno, Hiroko Kurosaki, Toshitaka Masutani, Hiroshi Inoue

**Affiliations:** The Second Department of Internal Medicine, University of Toyama, 2630 Sugitani, Toyama 930-0194, Japan

## Abstract

We examined whether hemorheology and platelet function are affected in essential hypertensives (EHTs) of the World Health Organization stage I when complicated with metabolic syndrome (Mets). In 156 untreated EHTs, blood viscosity and platelet surface markers were determined. Blood viscosity was significantly elevated in 54 subjects with Mets compared with 102 subjects without Mets. Hematocrit and plasma viscosity increased in the group with Mets, although red blood cell rigidity index “*k*” did not differ between groups. As a whole group, blood viscosity correlated positively with hematocrit and plasma viscosity. Additionally, plasma viscosity correlated positively with plasma leptin, triglyceride, homeostasis model assessment index, C-reactive protein, and plasma fibrinogen, but negatively with high-density lipoprotein cholesterol. In contrast, no differences were seen in platelet surface markers between groups. In conclusion, EHTs of the early stage complicated with Mets are characterized by increased blood viscosity due to hemoconcentration and increased plasma viscosity.

## 1. Introduction

Blood viscosity is one of the known determinants of vascular resistance and shear stress in the cardiovascular system, as shown by the Poiseuille equation [1]. Activation of platelets results in platelet aggregation and release of adenosine diphosphate (ADP), 5-hydroxytryptamine, thromboxane A_2_, and platelet-derived growth factor, thereby contributing to thrombus formation, vascular structural changes, and atherosclerosis [2–4]. Accordingly, increase in blood viscosity, platelet activation, or both is presumed to play a part in the pathogenesis of hypertension and other cardiovascular diseases.

 In 1999, metabolic syndrome (Mets) was first defined as a cluster of cardiovascular risk factors including obesity, impaired glucose tolerance, elevated blood pressure, and abnormalities of lipid metabolism [5]. Recently, epidemiologic studies have shown that patients with Mets have higher cardiovascular mortality and morbidity than the general population [6, 7]. The increased prevalence of cardiovascular diseases in Mets may be explained in part by abnormalities of blood viscosity or platelet function. In fact, increased blood viscosity and increased platelet reactivity are often found in patients with Mets [8–11]. Furthermore, it is well known that patients with essential hypertension also have increased blood viscosity [12, 13] and activated platelets [2, 14] and are often complicated with Mets [15]. However, it has not yet been elucidated how blood rheology and platelet functions are modulated in essential hypertensives (EHT) classified as stage I of the World Health Organization (WHO) severity score of hypertension [16] when complicated with Mets. In the present study, therefore, blood viscosity, platelet function, and factors possibly affecting these two indices were compared between early-stage EHT complicated with and without Mets. 

## 2. Methods

### 2.1. Subjects

 The study design was approved by the Ethics Committee at the University of Toyama. Untreated 156 EHTs (90 men, age  55 ± 12  years) were consecutively enrolled into the study after obtaining informed consent. The diagnosis of hypertension was made on the basis of a sitting diastolic blood pressure measured with a sphygmomanometer more than 90 mmHg and/or systolic blood pressure more than 140 mmHg on three separate occasions over a 4-week period. All subjects met the criteria for stage I on the WHO severity score of hypertension [16]. Of 66 female patients, 46 (70%) were postmenopausal, and none were on hormone replacement therapy. We excluded patients having macroalbuminuria (i.e., urinary albumin >300 mg/day), impaired renal function (i.e., creatinine clearance <60 mL/min/1.73 m^2^), secondary hypertension, diabetes mellitus, clinically evident cardiovascular diseases, or anemia. All medications that might affect blood viscosity or platelet function, for example, antiplatelet agents and nonsteroidal anti-inflammatory drugs, were discontinued at least 4 weeks prior to study commencement.

### 2.2. Study Protocol

 Mets was diagnosed following the Japanese diagnostic criteria [17] if patients had visceral obesity defined as waist circumference ≧85 and 90 cm in men and women, respectively, and at least one of the following risk factors: (1) fasting blood glucose ≧110 mg/dL and (2) plasma triglyceride ≧150 mg/dL and/or high density lipoprotein (HDL) cholesterol <40 mg/dL. Because various diagnostic criteria for Mets exist, IDF definition [18], one of the international criteria for Mets, was also employed.

Blood viscosity and platelet function were determined at the outpatient clinic in the morning. Patients were instructed not to take food, alcohol, caffeine, or cigarettes within the 12-hour period before the measurements. A venous catheter was inserted into the antecubital vein, and following 30 min supine rest, venous blood was collected gently without vein occlusion. Blood samples for determination of blood viscosity and platelet function were collected in tubes containing potassium EDTA and 0.38% sodium citrate, respectively, as anticoagulants. Blood samples were also taken for determination of parameters affecting hemorheology and platelet function [14, 19–22]. These included (1) hematocrit, (2) biochemical indices of glucose and lipid metabolism, (3) high-sensitive C-reactive protein (HS-CRP) as a marker of inflammation, and (4) plasma levels of leptin and fibrinogen. Subsequently, 24-hour urine collections were obtained for measurement of creatinine clearance and urinary albumin excretion.

For the hemorheological measurements, whole blood viscosity and plasma viscosity were determined using a falling ball microviscometer (AMVn-200, Anton Paar, Austria). This instrument comprises a glass capillary tube of 0.16 mm internal diameter containing a metallic ball of 0.15 mm diameter. Fluid viscosity is determined by the falling time of the ball in the tube inclined at 70 degrees filled with blood or plasma at 20°C [23, 24]. In this setting, fluid viscosity is measured at a high shear rate between 2500 and 3000 s^−1^, which allows measurement of blood viscosity in a completely disaggregated structure. In such a situation, it is theoretically considered that blood viscosity depends mainly on three parameters including plasma viscosity, red blood cell (RBC) rigidity, and hematocrit [24]. RBC rigidity was also evaluated using the RBC rigidity index “*k*” calculated according to the Quemada equation [25].

For determination of platelet functions, platelet surface markers were analyzed using whole-blood flow cytometry (EPICS XL, Coulter, Miami, FL, USA) [26]. A 5 *μ*L sample of citrated blood was diluted in 50 *μ*L of 10 mM HEPES buffer and mixed with vehicle or 1.0 *μ*M ADP as a platelet agonist. Then, 5 *μ*L of fluorescein isothiocyanate-conjugated anti-fibrinogen polyclonal antibody (Beckman Coulter, Inc., Miami, FL, USA), or phycoerythrin-conjugated anti-P-selectin monoclonal antibody (Beckman Coulter, Inc., Miami, FL, USA) was added, and the ratios of platelet fibrinogen binding and P-selectin expression were counted in 5000 platelets. In 9 healthy volunteers (5 men and 4 women), reproducibility of blood viscosity, plasma viscosity, and platelet surface markers was determined on 4 occasions at 1 week intervals. The intra subject coefficient of variance for these measurements was less than 5%.

Biochemical parameters were measured using conventional laboratory techniques. Insulin resistance was estimated according to the homeostasis model assessment (HOMA) as follows: HOMA index of insulin resistance (HOMA-IR) = fasting glucose (mg/dL) × fasting plasma insulin (*μ*IU/mL)/405. Plasma leptin levels were determined using commercial ELISA kits (Cayman Chemical, Ann Arbor, MI, USA).

### 2.3. Data Analyses

 Data are presented as mean ± standard deviation (SD). Comparisons were made between EHT with and without Mets using Student's *t*-test or chi-square (*χ* ^2^) test. In all patients, associations of blood viscosity or plasma viscosity with the factors possibly affecting these two indices were evaluated using Pearson's correlation. A *P* value less than 0.05 was considered statistically significant. 

## 3. Results

 Mets was present in 54 patients based on the Japanese criteria and in 55 based on the IDF criteria. Clinical characteristics, hemorheological indices, and platelet functions were similar between the two groups of patients with Mets (data not shown); therefore, the data analyses using the Japanese criteria of Mets are presented herein.

### 3.1. Clinical Characteristics

Due to the study design, body mass index and waist circumference were greater in patients with Mets than in those without Mets (Table 1). The proportion of patients with impaired glucose tolerance or dyslipidemia was also greater in patients with Mets than in those without Mets. No significant differences were seen in age, gender, prevalence of familial clustering of hypertension and menopause, and proportion of smokers between the two groups. Blood pressure at the outpatient clinic tended to be higher in patients with Mets than in those without Mets, although the difference was not statistically significant (Table 1).

### 3.2. Hemorheological Measurements and Platelet Functions (Table 2)

Blood viscosity was significantly higher in patients with Mets than in those without Mets. Both plasma viscosity and hematocrit were higher in patients with Mets compared with those without Mets, although RBC rigidity index “*k*” did not differ between groups. In contrast, no difference was seen in platelet function, estimated by fibrinogen binding and P-selectin expression, between groups.

### 3.3. Biochemical Variables (Table 3)

Due to the study design, serum levels of triglyceride, HOMA-IR, and HS-CRP were higher in patients with Mets than in those without Mets. By contrast, HDL-cholesterol levels were significantly lower in patients with Mets. Plasma leptin levels were higher in EHT with Mets compared with those without Mets, but no difference was seen in plasma fibrinogen between groups.

### 3.4. Factors Associated with Blood Viscosity or Plasma Viscosity

In the group of all the patients, blood viscosity correlated significantly to body mass index (*r* = 0.301, *P* < 0.001) and waist circumference (*r* = 0.230, *P* = 0.004), although it did not correlate to systolic blood pressure, diastolic blood pressure, or pulse rate (*r* = −0.031, 0.048, or 0.025 for each, ns). In addition, blood viscosity correlated positively with hematocrit (*r* = 0.757, *P* < 0.001) and also with plasma viscosity (*r* = 0.303, *P* < 0.001). Plasma viscosity correlated positively with HS-CRP, plasma leptin, serum triglyceride, HOMA-IR, and plasma fibrinogen, but negatively with serum HDL-cholesterol level (Table 4).

## 4. Discussion

The major findings of the present study are as follows. Firstly, whole blood viscosity was higher in EHT with Mets than in those without Mets. Likewise, blood viscosity was correlated with indices reflecting visceral fat mass such as waist circumference, but not with systemic hemodynamics in EHT. Secondly, no difference was seen in platelet function between the two groups. Therefore, in the early-stage EHT complicated with Mets, the hemorheological profile is altered as reported previously [9, 10], but platelet function is unaltered.

Blood viscosity measured by microviscometer depends mainly on plasma viscosity, RBC rigidity, and hematocrit [25]. Plasma viscosity is also affected by dyslipidemia, insulin resistance, inflammation, and fibrinogen [8, 19–21]. Additionally, abnormal secretions of adipocytokines can enhance cytokine productions and inflammation [22], possibly resulting in increased plasma viscosity in patients with Mets. In this study, elevated hematocrit and plasma viscosity were observed in EHT with Mets compared with those without Mets, although no difference was seen in RBC rigidity. As a whole group, blood viscosity correlated positively with hematocrit and plasma viscosity. As for the above-mentioned factors possibly affecting plasma viscosity, increased levels of plasma leptin, triglyceride, HOMA-IR, and serum HS-CRP and decreased level of HDL-cholesterol were detected in EHT with Mets. Moreover, plasma viscosity correlated positively with plasma leptin, serum triglyceride, HOMA-IR, HS-CRP, and plasma fibrinogen, but negatively with serum HDL-cholesterol. From these results, the early-stage EHTs with Mets are characterized by increased blood viscosity due to hemoconcentration and increased plasma viscosity. Furthermore, increased plasma viscosity can be attributed to the alterations in serum leptin, glucose, and lipid metabolism, inflammation, or plasma fibrinogen, all of which are often encountered in Mets.

Mets is characterized by disturbed secretion of adipocytokines from visceral fat deposits, for instance, increased secretion of leptin and decreased secretion of adiponectin. Increased secretion of leptin can stimulate sympathetic nervous system centrally [27]. On the other hand, decreased secretion of adiponectin can induce insulin resistance, and the subsequent hyperinsulinemia can enhance sympathetic nervous activity. Therefore, these combined effects can lead to peripheral vasoconstriction, thereby moving intravascular fluid to the interstitial space [28]. Indeed, the above sequence of events may have led to hemoconcentration in patients with Mets in this study, as evidenced by levels of plasma leptin, HOMA-IR, and hematocrit.

In contrast to blood viscosity, no difference was seen in platelet function between the early-stage EHTs with Mets and without Mets in the present study. Our previous study demonstrated that platelet function was influenced by sampling conditions (resting *versus* stress) and severity of hypertension (WHO classification) in EHT [2]. In the present study, the measurements were performed at rest only in EHT of the WHO stage I. Accordingly, further studies are required under different conditions and in EHT of more advanced stages of hypertension to determine influences of Mets on platelet function more clearly. The present study, however, demonstrated that platelet function is not affected by Mets in EHT without cardiovascular complications.

### 4.1. Study Limitations

The present study has several limitations. Firstly, this study was a cross-sectional study. Therefore, further longitudinal studies will be required to explore the long-term influences of abnormal hemorheological properties on systemic blood pressure and cardiovascular complications in EHT with Mets. Secondly, the present study did not include normotensive subjects with and without Mets. These two groups should have been included to draw a definite conclusion concerning the additive effects of hypertension and Mets on blood rheology and platelet function. Thirdly, the effects of therapeutic interventions on abnormalities in blood hemorheology in EHT with Mets also need to be studied. Alpha-1 blockers or calcium antagonists could be recommended because they reduce blood viscosity in EHT [29–31]. Finally, EHT of more advanced stages should be studied, as the subjects in this study were confined to those without cardiovascular complications.

Although limited for these reasons, the present study showed that presence of Mets is associated with an increase in blood viscosity, without affecting platelet function in EHT of the WHO stage I. These results suggest that therapeutic interventions against hemorheological abnormalities need to be started earlier in order to prevent cardiovascular complications in EHT with Mets.

## Figures and Tables

**Table 1:** Comparison of clinical characteristics between essential hypertensives with and without metabolic syndrome.

Variables	Metabolic syndrome		*P* value
	(−)	(+)	
Number	102	54	
Age (years)	55 ± 12	55 ± 12	0.935
Sex (men/women)	56/46	34/20	0.332
Menopause in female patients	30 (65)	16 (80)	0.229
Body mass index (kg/m^2^)	23.5 ± 3.7	27.7 ± 2.9	<0.001
Waist circumference (cm)	81 ± 8	89 ± 11	<0.001
Family history of hypertension	64 (63)	34 (63)	0.979
Current smoker	13 (13)	11 (20)	0.209
Impaired glucose tolerance	3 (3)	29 (54)	<0.001
Dyslipidemia	5 (5)	30 (56)	<0.001
Systolic blood pressure (mmHg)	153 ± 20	160 ± 21	0.051
Diastolic blood pressure (mmHg)	93 ± 14	97 ± 15	0.101
Pulse rate (beats/minutes)	72 ± 13	70 ± 10	0.311

Values are mean ± SD or number (%) of patients. Impaired glucose tolerance was defined as fasting blood glucose ≥ 110 mg/dL, and dyslipidemia as plasma triglyceride ≥ 150 mg/dL and/or HDL cholesterol <40 mg/dL.

**Table 2:** Comparison of hemorheological variables, red blood cell (RBC) rigidity index “*k*”, hematocrit and platelet function between essential hypertensives with and without metabolic syndrome.

Variables	Metabolic syndrome		*P* value
	(−)	(+)	
Blood viscosity (mPa·S)	4.01 ± 0.42	4.33 ± 0.52	<0.001
Plasma viscosity (mPa·S)	1.73 ± 0.07	1.76 ± 0.07	0.019
RBC rigidity index “*k*”	1.73 ± 0.11	1.76 ± 0.13	0.237
Hematocrit (%)	39.4 ± 3.6	40.8 ± 3.6	0.025
Fibrinogen binding			
Vehicle (%)	24.2 ± 8.1	26.1 ± 6.6	0.135
ADP 1.0 *μ*M (%)	61.5 ± 19.1	64.2 ± 18.5	0.389
P-selectin expression			
vehicle (%)	15.3 ± 6.4	14.7 ± 4.8	0.567
ADP 1.0 *μ*M (%)	48.4 ± 17.8	49.0 ± 16.9	0.848

Values are mean ± SD. ADP: adenosine diphosphate.

**Table 3:** Comparison of biochemical variables between essential hypertensives with and without metabolic syndrome.

Variables	Metabolic syndrome		*P* value
	(−)	(+)	
Total cholesterol (mg/dL)	191 ± 35	207 ± 50	0.025
Triglyceride (mg/dL)	84 ± 43	148 ± 75	<0.001
HDL-cholesterol (mg/dL)	58 ± 14	46 ± 11	<0.001
Blood glucose (mg/dL)	94 ± 9	102 ± 11	<0.001
HOMA-IR	1.0 ± 0.6	2.3 ± 2.4	<0.001
High-sensitive C-reactive protein (mg/dL)	0.07 ± 0.12	0.17 ± 0.30	0.003
Plasma fibrinogen (mg/dL)	236 ± 49	242 ± 57	0.513
Plasma leptin (*μ*g/mL)	6.1 ± 5.2	8.6 ± 5.9	0.008

Values are mean ± SD. HDL: high-density lipoprotein; HOMA-IR: homeostasis model assessment index of insulin resistance.

**Table 4:** Correlations between plasma viscosity and biochemical variables.

Variables	*r*	*P* value
Triglyceride	0.236	0.003
HDL-cholesterol	−0.204	0.011
Blood glucose	0.072	0.374
HOMA-IR	0.219	0.006
High-sensitive C-reactive protein	0.318	<0.001
Plasma fibrinogen	0.382	<0.001
Plasma leptin	0.263	<0.001

Abbreviations are as in Table 3.

## Abbreviations

EHT: Essential hypertensives
Mets: Metabolic syndrome
ADP: Adenosine diphosphate
WHO: World Health Organization
HDL: High-density lipoprotein
HS-CRP: High-sensitive C-reactive protein
RBC: Red blood cell
HOMA-IR: Homeostasis model assessment index of insulin resistance.
